# Supplementary material for: Compression-induced expression of glycolysis genes in CAFs correlates with EMT and angiogenesis gene expression in breast cancer
Source: Commun Biol. 2019 Aug 14;2:313. doi: 10.1038/s42003-019-0553-9 (PMC6694123; doi:10.1038/s42003-019-0553-9)
Supplement: Supplementary file 1 — Supplementary Information [file 42003_2019_553_MOESM1_ESM.pdf]

Supplementary Information

Supplementary Figures

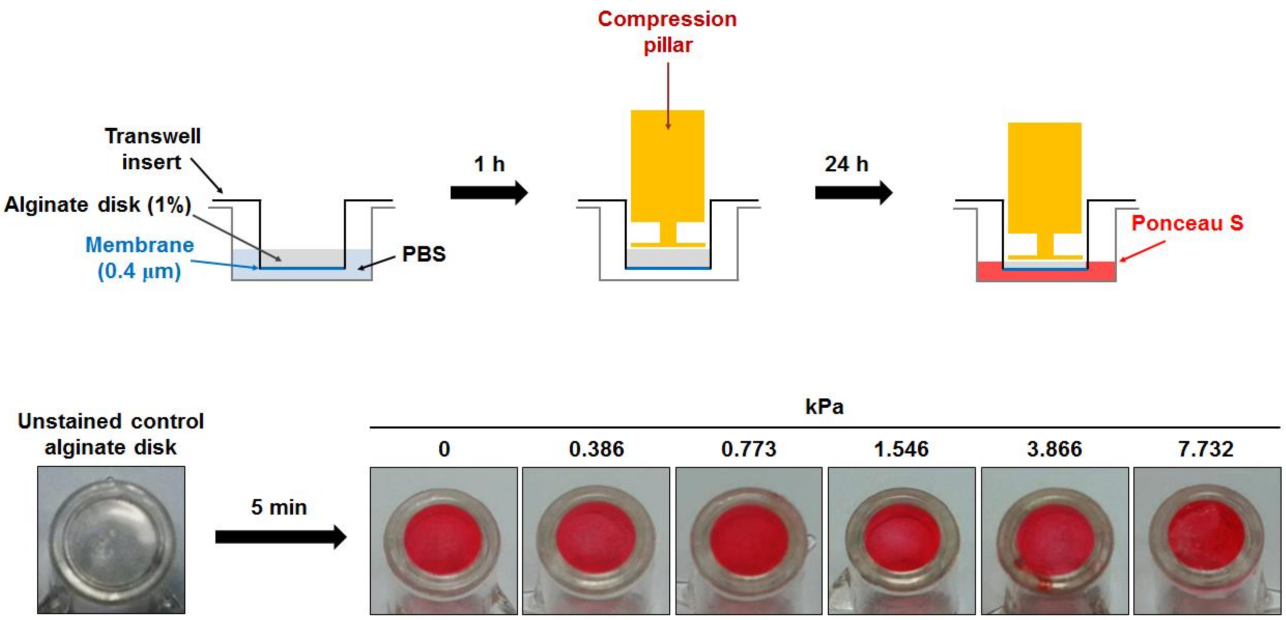

Supplementary Figure 1. Dye diffusion rate in the alginate disks exposed to different degrees of compressive stress. Alginate disks (1%) were formed on transwell membrane (0.4 μm). After being equilibrated with PBS for 1h at 37℃, the alginate disks were exposed to different degrees of compressive stress for 24h at 37℃. For dye diffusion assay, the PBS in the lower chamber was replaced with Ponceau S and then incubated for 5 min at 37℃.

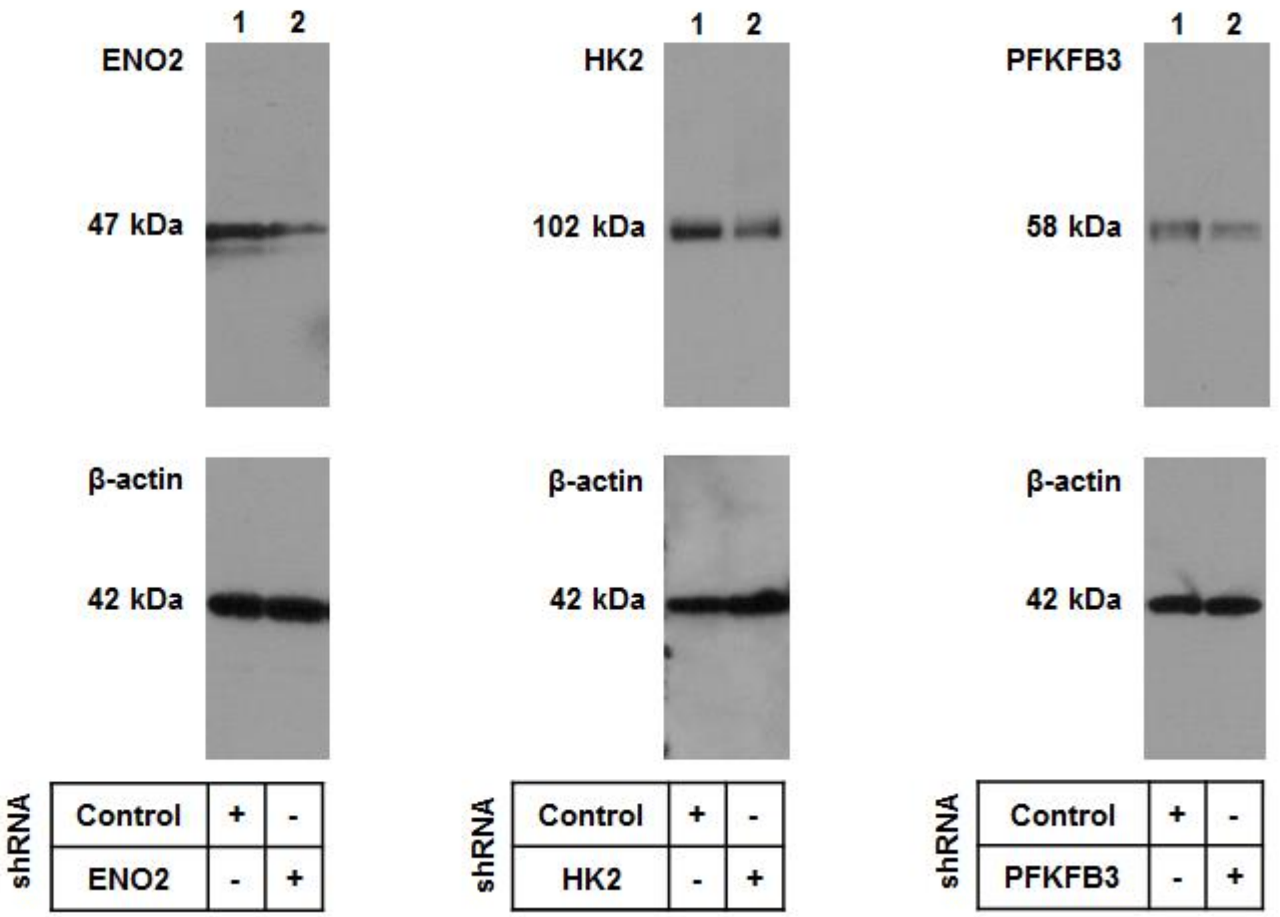

Supplementary Figure 2. The knockdown of ENO2, HK2, and PFKFB3 expression in CAF cells. CAF cells were transfected with control shRNA or the shRNAs against *ENO2*, *HK2*, or *PFKFB3* gene. β-actin was detected on different membranes since its size was similar to others except for HK2. The images were brighten and cropped in the main text, and correspond to Figure 4E.

Lane	1	2	3	4	5	6
CS (kPa)	0	0.773	3.886	0	0.773	3.886
SP600125	-	-	-	+	+	+

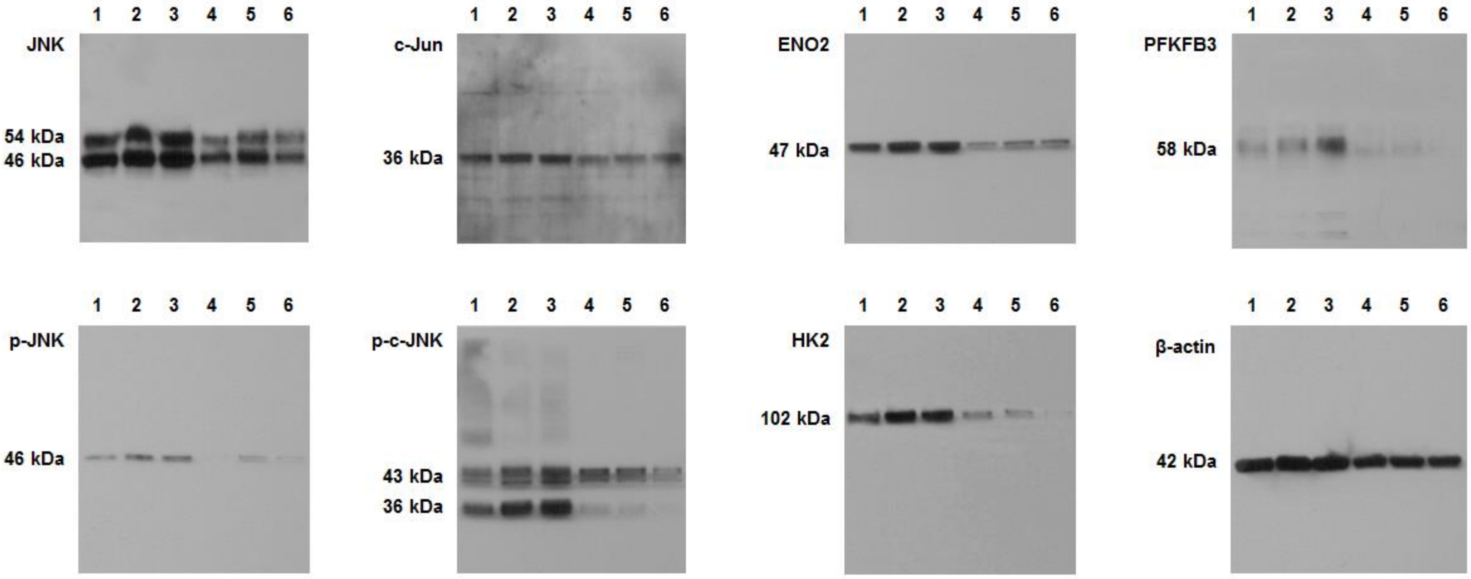

Supplementary Figure 3. The protein expression of ENO2, HK2, and PFKFB3 genes in the CAF cells exposed to compressive stress and/or treated with c-Jun inhibitor. CAF cells were pre-treated with DMSO (lane 1 to 3) or 50 uM of SP600125, c-Jun phosphorylation inhibitor (lane 4 to 6) for 1 day. For compression, the CAF cells pre-treated with DMSO or SP600125 were harvested, embedded into 1% alginate disk, and then exposed to 0 (lane 1 and 4), 0.773 (lane 2 and 5), or 3,886 (lane 3 and 6) kPa for 1 day. After compression, the CAF cells were recovered from the alginate disk by depolymerization with 40 mM EDTA. The images were cropped in the main text and correspond to Figure 5D.

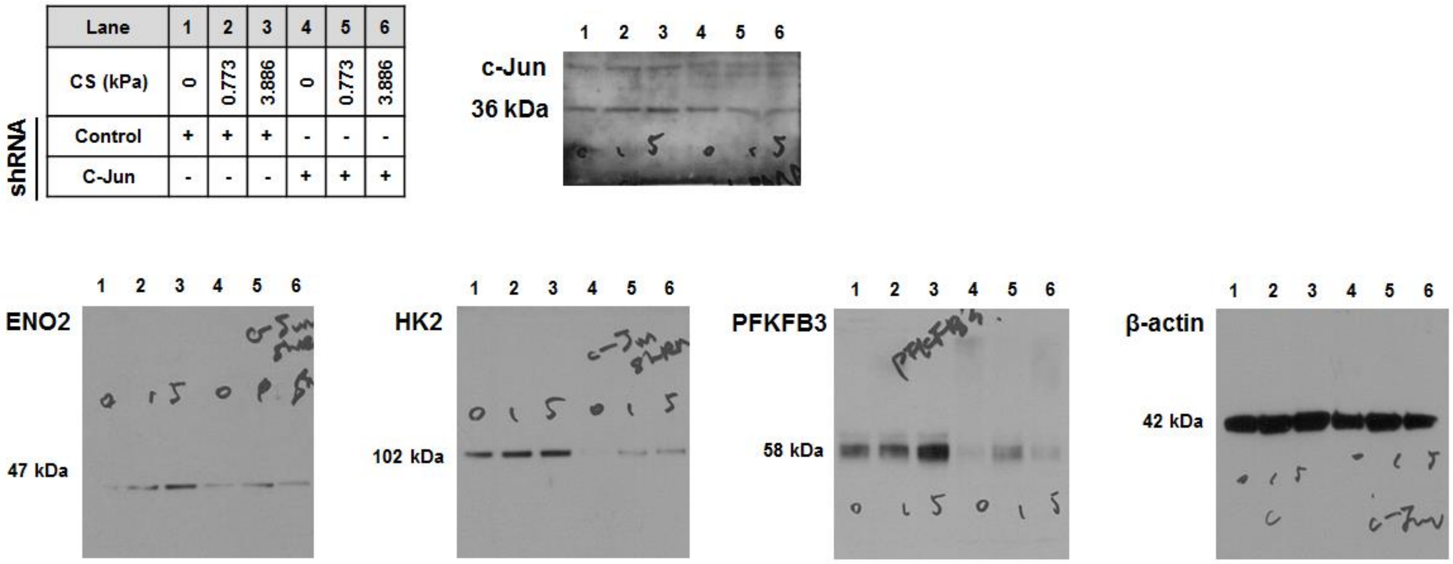

Supplementary Figure 4. The compression-induced expression of ENO2, HK2, and PFKFB3 in the c-Jun knockdown CAF cells. CAF cells were transfected with control shRNA (lane 1 to 3) or c-Jun shRNA (lane 4 to 6). For compression, the CAF cells transfected with control or c-Jun shRNA were harvested, embedded into 1% alginate disk, and then exposed to 0 (lane 1 and 4), 0.773 (lane 2 and 5), or 3,886 (lane 3 and 6) kPa for 1 day. After compression, the CAF cells were recovered from the alginate disk by depolymerization with 40 mM EDTA. The images were cropped in the main text and correspond to Figure 5F.

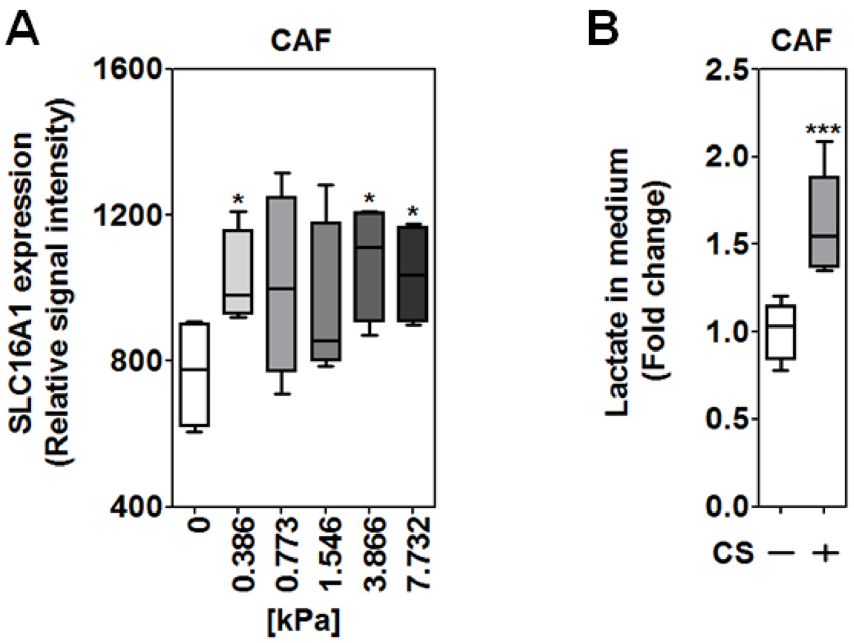

Supplementary Figure 5. A) The compression-induced upregulation of *SLC16A1* gene in CAF cells (n=4 independent experiment). B) Lactate assay with the CM from CAF cells with or without compression (n=7 independent experiment). For compression, CAF cells were exposed to 0.773 kPa for 1 day. Error bars and p-values were determined by Whiskers (Min to Max) and unpaired two-tailed t-test, respectively. The source data are provided as Supplementary Data 1.

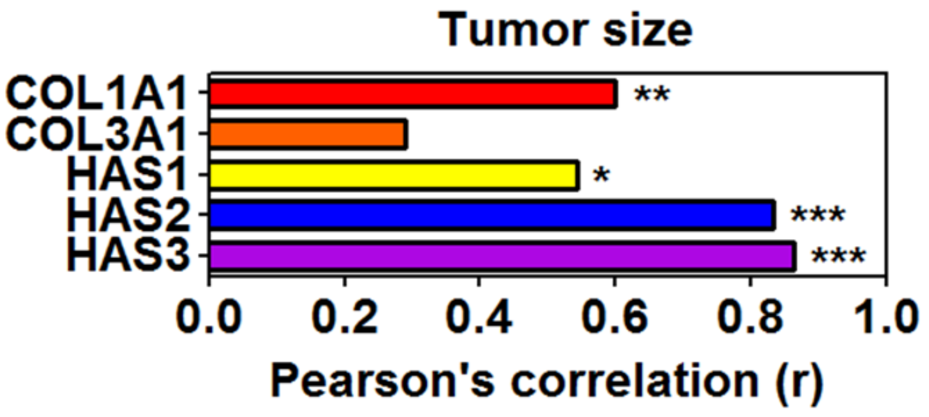

Supplementary Figure 6. The expression correlation between tumor size and compressive stress markers in breast cancer patient tissues. Pearson's correlation coefficient was analyzed from 20 cases of breast cancer patient tissues using Prisim 6 for Windows (GraphPad Software, Inc.; La Jolla, CA). Asterisks were used to indicate p values: one for $p < 0.05$, two for $p < 0.01$, and three for $p < 0.001$. Source data are provided as Supplementary Data 1.

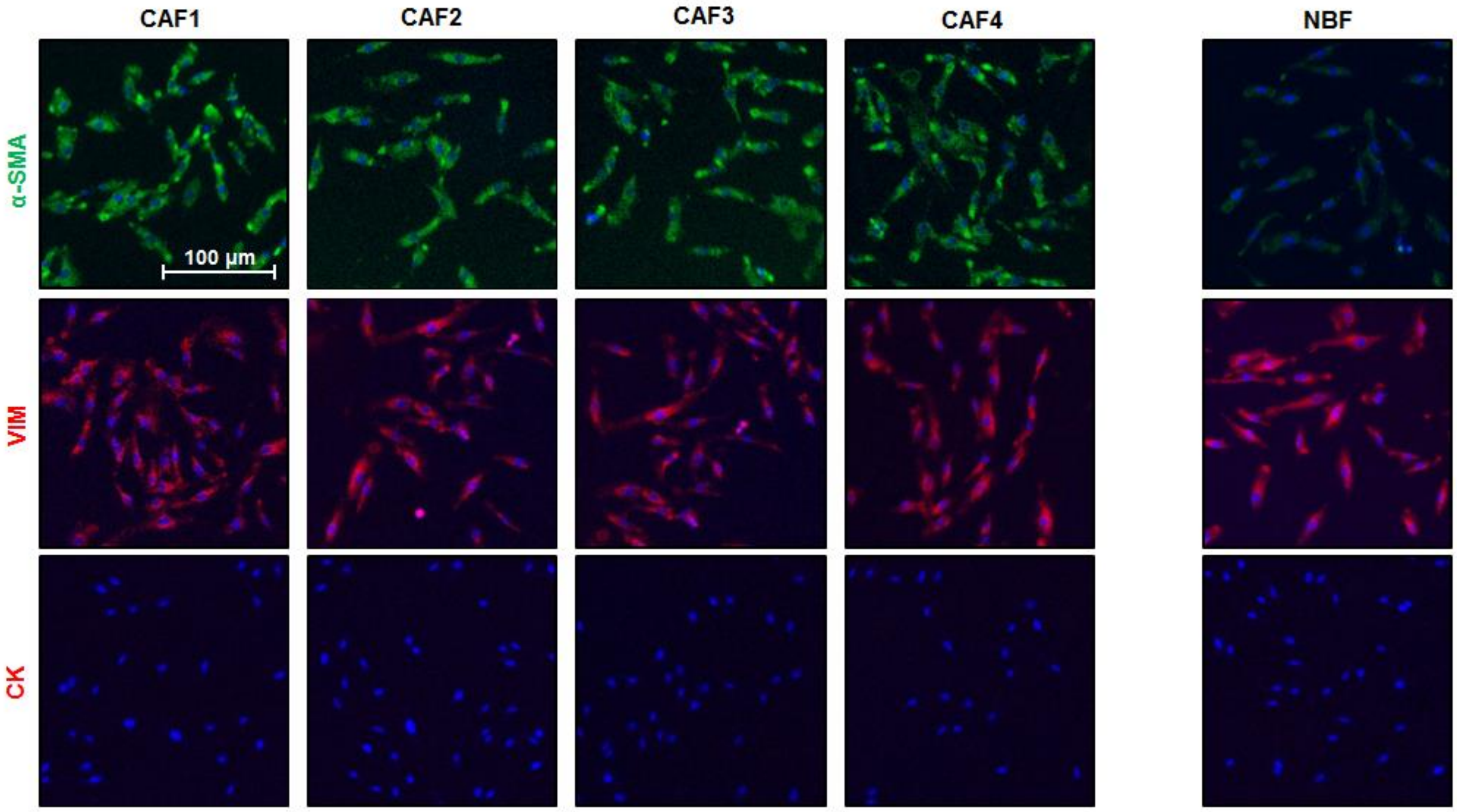

Supplementary Figure 7. The immunofluorescence characterization of cancer-associated fibroblasts. The natures of primary cancer-associated fibroblasts (CAFs) were confirmed by immunofluorescence characterization using antibodies against vimentin (VIM, Abcam; Cambridge, UK), alpha-smooth muscle actin (α-SMA, Santa Cruz Biotechnology, Dallas, TX), and cytokeratin (CK, Dako; Glostrup, Denmark). Compared to Normal breast fibroblast (NBF), CAFs showed a higher signal intensity of α-SMA (a marker for CAFs). Both NBF and CAFs were VIM (a marker for fibroblast)-positive.

Supplementary tables

Supplementary Table 1.

Gene name	Strand	Sequence (5' to 3')	Tm (°C)	Use
ENO2	Forward	GAAGAAAAGGCCTGCAACTG	55.4	mRNA expression analysis
	Reverse	CCAGGTCAGCAATGAATGTG	55.0	
HK2	Forward	TCTATGCCATCCCTGAGGAC	57.3	
	Reverse	TCTCTGCCTTCCACTCCACT	59.2	
PFKFB3	Forward	CTTGTCGCTGATCAAGGTGA	55.8	
	Reverse	TTCTGCTCCTCCACGAACTT	57.0	
GAPDH	Forward	TGCACCACCAACTGCTTAGC	55.9	
	Reverse	GGCATGGACTGTGGTCATGAG	57.3	
SDHA	Forward	TGGGAACAAGAGGGCATCTG	57	
	Reverse	CCACCACTGCATCAAATTCATG	57.3	
HPRT1	Forward	TGACACTGGCAAAACAATGCA	57.1	
	Reverse	GGTCCTTTTCACCAGCAAGCT	57	
ENO2-S2	Forward	GAGAGGAAAAGGGACCCAAG	56.4	Chromatin immunoprecipitation
	Reverse	CCCACACCTGTCCTTTCCTA	57.7	
HK2-S1	Forward	TTTCCATGGCAATGACTCAA	52.6	
	Reverse	ACATCAGGAGCAGAGGCACT	59.8	
HK2-S2	Forward	CTGCAACTCCAGGAGAGGAC	59.1	
	Reverse	AGGCTCCTGAGTCCCTGTTT	59.6	
PFKFB3-S1	Forward	GCTGCAGTGAGCTATGATCG	57.2	
	Reverse	AGGACGAGACCTGAGGGAAT	58.7	
