# Supplementary material for: Compression-induced expression of glycolysis genes in CAFs correlates with EMT and angiogenesis gene expression in breast cancer
Source: Commun Biol. 2019 Aug 14;2:313. doi: 10.1038/s42003-019-0553-9 (PMC6694123; doi:10.1038/s42003-019-0553-9)
Supplement: Supplementary file 2 — Description of Additional Supplementary Files [file 42003_2019_553_MOESM2_ESM.pdf]

Description of additional supplementary items

Supplementary Data 1

An excel document containing the data used for the making of each figure and supplementary figure.

- Sheet 1: Figure 1C
- Sheet 2: Figure 1D
- Sheet 3: Figure 2
- Sheet 4: Figure 3A
- Sheet 5: Figure 3B
- Sheet 6: Figure 4A
- Sheet 7: Figure 4B
- Sheet 8: Figure 4C
- Sheet 9: Figure 4D
- Sheet 10: Figure 4E
- Sheet 11: Figure 4F
- Sheet 12: Figure 5A
- Sheet 13: Figure 5B
- Sheet 14: Figure 5C
- Sheet 15: Figure 5E
- Sheet 16: Figure 5G
- Sheet 17: Figure 6A
- Sheet 18: Figure 6B
- Sheet 19: Supplementary Figure 2A
- Sheet 20: Supplementary Figure 2B
- Sheet 21: Figure 7A
- Sheet 22: Figure 7B
- Sheet 23: Figure 7C
- Sheet 24: Figure 7D

- Sheet 25: Figure 7E
- Sheet 26: Figure 7F
- Sheet 27: Figure 7G
- Sheet 28: Figure 7H
- Sheet 29: Figure 7I
- Sheet 30: Supplementary Figure 3
- Sheet 31: Figure 8C
- Sheet 32: Figure 9A
- Sheet 33: Figure 9B
- Sheet 34: Figure 9C
- Sheet 35: Figure 9D

Supplementary Data 2

An excel document containing the DAVID functional annotation results used for the making of Figure 2.

- Sheet 1: Upregulated genes of BT-474 cells
- Sheet 2: Downregulated genes of BT-474 cells
- Sheet 3: Upregulated genes of MCF7 cells
- Sheet 4: Downregulated genes of MCF7 cells
- Sheet 5: Upregulated genes of SK-BR-3 cells
- Sheet 6: Downregulated genes of SK-BR-3 cells
- Sheet 7: Upregulated genes of MDA-MB-231 cells
- Sheet 8: Downregulated genes of MDA-MB-231 cells
- Sheet 9: Upregulated genes of CAF1 cells
- Sheet 10: Downregulated genes of CAF1 cells
- Sheet 11: Upregulated genes of CAF2 cells
- Sheet 12: Downregulated genes of CAF2 cells

- Sheet 13: Upregulated genes of CAF3 cells
- Sheet 14: Downregulated genes of CAF3 cells
- Sheet 15: Upregulated genes of CAF4 cells
- Sheet 16: Downregulated genes of CAF4 cells
