# Supplementary material for: Compression-induced expression of glycolysis genes in CAFs correlates with EMT and angiogenesis gene expression in breast cancer
Source: Commun Biol. 2019 Aug 14;2:313. doi: 10.1038/s42003-019-0553-9 (PMC6694123; doi:10.1038/s42003-019-0553-9)
Supplement: Supplementary file 6 — Reporting Summary [file 42003_2019_553_MOESM6_ESM.pdf]

nature research

Corresponding author(s): Baek Gil Kim

Last updated by author(s): Jul 11, 2019

Reporting Summary

Nature Research wishes to improve the reproducibility of the work that we publish. This form provides structure for consistency and transparency in reporting. For further information on Nature Research policies, see Authors & Referees and the Editorial Policy Checklist.

Statistics

For all statistical analyses, confirm that the following items are present in the figure legend, table legend, main text, or Methods section.

n/a	Confirmed	
☐	☒	The exact sample size (*n*) for each experimental group/condition, given as a discrete number and unit of measurement
☐	☒	A statement on whether measurements were taken from distinct samples or whether the same sample was measured repeatedly
☐	☒	The statistical test(s) used AND whether they are one- or two-sided<br>*Only common tests should be described solely by name; describe more complex techniques in the Methods section.*
☒	☐	A description of all covariates tested
☒	☐	A description of any assumptions or corrections, such as tests of normality and adjustment for multiple comparisons
☐	☒	A full description of the statistical parameters including central tendency (e.g. means) or other basic estimates (e.g. regression coefficient) AND variation (e.g. standard deviation) or associated estimates of uncertainty (e.g. confidence intervals)
☒	☐	For null hypothesis testing, the test statistic (e.g. *F*, *t*, *r*) with confidence intervals, effect sizes, degrees of freedom and *P* value noted<br>*Give P values as exact values whenever suitable.*
☒	☐	For Bayesian analysis, information on the choice of priors and Markov chain Monte Carlo settings
☒	☐	For hierarchical and complex designs, identification of the appropriate level for tests and full reporting of outcomes
☒	☐	Estimates of effect sizes (e.g. Cohen's *d*, Pearson's *r*), indicating how they were calculated

Our web collection on statistics for biologists contains articles on many of the points above.

Software and code

Policy information about availability of computer code

Data collection	cBioPortal for Cancer Genomics, The Human Protein Atlas, Kaplan-Meier Plotter, Amigo-Gene Ontology
Data analysis	DAVID Functional Annotation Bioinformatics Microarray Analysis, Kaplan-Meier Plotter, Prism, Circos

For manuscripts utilizing custom algorithms or software that are central to the research but not yet described in published literature, software must be made available to editors/reviewers. We strongly encourage code deposition in a community repository (e.g. GitHub). See the Nature Research guidelines for submitting code & software for further information.

Data

Policy information about availability of data

All manuscripts must include a data availability statement. This statement should provide the following information, where applicable:

- Accession codes, unique identifiers, or web links for publicly available datasets
- A list of figures that have associated raw data
- A description of any restrictions on data availability

All data supporting the findings of this study are available in Supplementary Information and Supplementary Data 1 and 2. Microarray raw data generated in study have been deposited into the GEO database (http://www.ncbi.nlm.nih.gov/geo/) under accession numbers GSE133134. Previously generated data analysed here are available in The Human Protein Atlas (version 18.1) and in METABRIC dataset in cBioportal. All patients donating the tissues had surgery at Severance Hospital of the Yonsei University Health System, South Korea.

Field-specific reporting

Please select the one below that is the best fit for your research. If you are not sure, read the appropriate sections before making your selection.

☒ Life sciences ☐ Behavioural & social sciences ☐ Ecological, evolutionary & environmental sciences

For a reference copy of the document with all sections, see nature.com/documents/nr-reporting-summary-flat.pdf

Life sciences study design

All studies must disclose on these points even when the disclosure is negative.

Sample size	Sufficient sample sizes were chosen for each experiment to determine whether the outcome was statistically significant.
Data exclusions	No data were excluded.
Replication	All experiments were at least triplicated.
Randomization	Randomly selected samples were allocated into experimental groups.
Blinding	Blinding was not implemented in this study.

Reporting for specific materials, systems and methods

We require information from authors about some types of materials, experimental systems and methods used in many studies. Here, indicate whether each material, system or method listed is relevant to your study. If you are not sure if a list item applies to your research, read the appropriate section before selecting a response.

Materials & experimental systems

n/a	Involved in the study	
☐	☒	Antibodies
☐	☒	Eukaryotic cell lines
☒	☐	Palaeontology
☒	☐	Animals and other organisms
☐	☒	Human research participants
☒	☐	Clinical data

Methods

n/a	Involved in the study	
☒	☐	ChIP-seq
☒	☐	Flow cytometry
☒	☐	MRI-based neuroimaging

Antibodies

Antibodies used	anti-vimentin (1:1000, ab20346, Abcam), anti-alpha smooth muscle actin (1:200, ab7817, Abcam), anti-cytokeratin (1:100, sc-32721, SantaCruz Biotechnology), anti-JNK (1:1000, sc-571, SantaCruz Biotechnology), anti-phospho-JNK (1:200, sc-6254, SantaCruz Biotechnology) , anti-c-Jun (1:1000, sc-1694, SantaCruz Biotechnology), anti-phospho-c-Jun (1:500, sc-822, SantaCruz Biotechnology), anti-ENO2 (1:1000, CSB-PA002348, Cusabio), anti-HK2 (1:1000, CSB-PA132121, Cusabio), anti-PFKFB3 (1:1000, CSB-PA017819GA01HU, Cusabio), HRP-conjugated anti-mouse (1:10000, GeneDepot, SA001), HRP-conjugated anti-rabbit (1:10000, SA002, GeneDepot)
Validation	All antibodies were validated by the suppliers.

Eukaryotic cell lines

Policy information about cell lines

Cell line source(s)	All cell lines were purchased from the Korean Cell Line Bank.
Authentication	All cell lines were authenticated using morphology and STR profiling.
Mycoplasma contamination	All cell lines were tested negative for mycoplasma contamination.
Commonly misidentified lines (See ICLAC register)	None

Human research participants

Policy information about studies involving human research participants

Population characteristics	20 female patients, ages between 37-81 years. Diagnosis for 17 patients IDC, NOS, 2 patients IDC, tubular, 1 patient IDC, mucinous focal. All patients were treated by mastectomy with axially lymph node dissection.
Recruitment	Of the patients having breast cancer surgery at Severance Hospital of Yonsei University Health System (and donating their tissues), the patients imaged by SWE were selected and classified into a low- or high-compression group.
Ethics oversight	The study protocol was approved by the Severance Hospital Ethics Committee.

Note that full information on the approval of the study protocol must also be provided in the manuscript.
